# Enhanced Bone Marrow Homing of Natural Killer Cells Following mRNA Transfection With Gain-of-Function Variant CXCR4^R334X^

**DOI:** 10.3389/fimmu.2019.01262

**Published:** 2019-06-05

**Authors:** Emily Levy, Robert Reger, Filip Segerberg, Melanie Lambert, Caroline Leijonhufvud, Yvonne Baumer, Mattias Carlsten, Richard Childs

**Affiliations:** ^1^National Heart Lung and Blood Institute, National Institutes of Health, Bethesda, MD, United States; ^2^The Department of Molecular Medicine, The George Washington University, Washington, DC, United States; ^3^Department of Medicine, Huddinge, Center for Hematology and Regenerative Medicine, Karolinska Institutet, Stockholm, Sweden

**Keywords:** NK cells, homing, mRNA transfection, CXCR4, immunotherapy, WHIM

## Abstract

Adoptive transfer of natural killer (NK) cells can induce remission in patients with relapsed/refractory leukemia and myeloma. However, to date, clinical efficacy of NK cell immunotherapy has been limited to a sub-fraction of patients. Here we show that steps incorporated in the *ex vivo* manipulation/production of NK cell products used for adoptive infusion, such as over-night IL-2 activation or cryopreservation followed by *ex vivo* expansion, drastically decreases NK cell surface expression of the bone marrow (BM) homing chemokine receptor CXCR4. Reduced CXCR4 expression was associated with dampened *in vitro* NK cell migration toward its cognate ligand stromal-derived factor-1α (SDF-1α). NK cells isolated from patients with WHIM syndrome carry gain-of-function (GOF) mutations in CXCR4 (CXCR4^R334X^). Compared to healthy donors, we observed that NK cells expanded from WHIM patients have similar surface levels of CXCR4 but have a much stronger propensity to home to BM compartments when adoptively infused into NOD-*scid* IL2Rgamma^null^ (NSG) mice. Therefore, in order to augment the capacity of adoptively infused NK cells to home to the BM, we genetically engineered *ex vivo* expanded NK cells to express the naturally occurring GOF CXCR4^R334X^ receptor variant. Transfection of CXCR4^R334X^-coding mRNA into *ex vivo* expanded NK cells using a clinically applicable method consistently led to an increase in cell surface CXCR4 without altering NK cell phenotype, cytotoxic function, or compromising NK cell viability. Compared to non-transfected and wild type CXCR4-coding mRNA transfected counterparts, CXCR4^R334X^-engineered NK cells had significantly greater chemotaxis toward SDF-1α *in vitro*. Importantly, expression of CXCR4^R334X^ on expanded NK cells resulted in significantly greater BM homing following adoptive transfer into NSG mice compared to non-transfected NK cell controls. Collectively, these data suggest up-regulation of cell surface CXCR4^R334X^ on *ex vivo* expanded NK cells via mRNA transfection represents a novel approach to improve homing and target NK cell-based immunotherapies to BM where hematological malignancies reside.

## Introduction

Natural killer (NK) cells play an important role in the body's defense against cancer and have proven to be powerful effectors against hematological tumor targets in pre-clinical studies (1). During the last decade, several published studies have demonstrated that adoptive infusion of mature short-term cytokine activated allogeneic NK cells can induce remission in subgroups of patients with relapsed/refractory leukemia and myeloma (2, 3). Clinical trials are currently evaluating the safety and efficacy of the adoptive infusion of *ex vivo* expanded NK cells (NCT02074657; NCT02271711). Advantages of *ex vivo* expansion include the possibility of utilizing repeated infusions of large numbers of these highly cytotoxic effector cells. Despite decades of promising preclinical studies, at present, only a relatively small proportion of patients treated with adoptive NK cell infusions have shown a clearly beneficial response (4, 5). NK cell immunotherapy may in part be limited by host factors such as tumor burden and aggressiveness, immune-mediated rejection of the infused cells, and the inhibitory effects of suppressive cell populations such as regulatory T cells (5–7). Deeper biological insights into these factors as well as the development of novel strategies to overcome these limitations are needed before the full therapeutic potential of NK cell immunotherapy can be realized in cancer patients.

A still relatively unexplored aspect of cellular cancer immunotherapy is the potential of the infused cells to home to the cancer-affected organ *in vivo* (8). For instance, the potential of adoptively infused NK cells to home to bone marrow (BM) compartments where hematological malignancies reside is likely critical to obtain clinically meaningful antitumor effects in patients with leukemia or myeloma. Consistent with this theory, a recent analysis by Bjorklund et al. showed that high expression levels of the BM homing chemokine receptor CXCR4 on the infused NK cells was associated with increased probability of objective response in patients with relapsed/refractory acute myeloid leukemia (AML) or high-risk myelodysplastic syndrome (MDS) (5). These and previously published data showing that CXCR4 expression is reduced on the NK cell surface shortly after activation with IL-2 (9) have led our group to hypothesize that modification of NK cells to express higher levels of CXCR4, including CXCR4 receptor variants associated with a gain-of-function (GOF), may represent a method to improve the clinical efficacy of adoptive NK cell immunotherapy for hematological malignancies.

There has been recent increased interest in the use of a number of different strategies to genetically modify NK cells to augment their anti-tumor potential (10). We have previously shown that mRNA electroporation can be used as an approach to efficiently transfect *ex vivo* expanded NK cells using the FDA approved MaxCyte platform (11). Therefore, we explored whether mRNA electroporation of CXCR4, including the WHIM syndrome naturally occurring GOF mutated variant CXCR4^R334X^ (12), could be utilized to improve NK cell *in vitro* migration toward its ligand stromal-derived factor-1α (SDF-1α) and *in vivo* homing to BM compartments in mice. We further sought to validate this strategy by testing the hypothesis that NK cells expanded *ex vivo* from WHIM patients with GOF CXCR4^R334X^ would have superior homing to the bone marrow compared to NK cells expressing wild-type CXCR4.

Here we show that commonly utilized methods to *ex vivo* activate and/or expand NK cells for adoptive infusion, such as over-night IL-2 activation or cryopreservation followed by *ex vivo* expansion, drastically decrease NK cell surface expression of CXCR4 compared to non-manipulated NK cells from PBMCs. This attenuation in receptor expression intensity was associated with a marked reduction in their capacity to migrate toward SDF-1α *in vitro*. Further, we show that NK cells expanded from WHIM patients have similar surface levels of CXCR4 but a much stronger propensity to home to BM compartments when adoptively infused into NOD-*scid* IL2Rgamma^null^ (NSG) mice. Finally, we show that *ex vivo* expanded human NK cells genetically engineered to express the GOF CXCR4^R334X^ receptor variant have significantly enhanced *in vitro* migration capacity toward SDF-1α and superior BM homing following adoptive transfer into NSG mice compared to non-transfected NK cell controls. Collectively, these data suggest introduction of CXCR4^R334X^ on the cell surface of *ex vivo* expanded NK cells via mRNA transfection represents a novel approach to improve their BM homing capacity and thereby augment NK cell-based immunotherapies against BM-residing malignancies such as leukemia and myeloma.

## Materials and Methods

### Cells Lines and Primary Cells

Peripheral blood mononuclear cells (PBMCs) from healthy donor blood were isolated using Lymphocyte Separation Medium (MP Biomedicals) (NIH protocol 99-H-0050 and 2006/229-31/3). NK cells were isolated by magnetic bead separation; either CD3 depleted and CD56 selected (Miltenyi) or by using an NK cell isolation kit (Miltenyi). NK cells were expanded for 14–18 days in G-Rex flasks (Wilson Wolf Manufacturing) with irradiated human EBV-LCL feeder cells at a ratio of one NK cell to 10 feeder cells (1:10 ratio). Cells were cultured in X-vivo 20 media (Lonza) containing 10% human AB serum (Sigma), 1% Glutamax (Gibco), and 500 U/ml IL-2. Fresh media was supplied to cells starting on day 5 of expansion, and then every 48 h until the cells were harvested for use in experiments. Cryopreserved WHIM PBMCs (NIH protocols NCT05-I-0213; NCT14-I-0185) were generously provided by Dr. David McDermott through an NIH Material Transfer Agreement in accordance with NIH human subject research policies. K562 and MOLM14 cell lines were obtained from ATCC. MM.1S was kindly provided by Dr. Irene Ghobrial at the Dana-Farber Cancer Institute. SMI-LCLs were established in the NHLBI/NIH by the Childs' lab. Cell lines were propagated in RPMI 1640 supplemented with 10% heat-inactivated FBS (Sigma-Aldrich).

### Antibodies and Flow Cytometry

Phenotype analysis were conducted antibodies targeting human CD56 (NCAM16.2), CD3 (UCHT1), CD57 (NK-1), CD2 (RPA2.10), DNAM-1 (DX11), NKp44 (p44-8), NKp30 (p30-15), NKp46 (9E2), TNF-α (Mab11), Granzyme B (Gb11), and IFN-γ (B27) from Becton Dickinson (BD) Biosciences (CA, USA). Antibodies targeting human CXCR4 (12G5), IgG2a isotype (MOPC173), KIR2DL/DS/2/3 (DX27), NKG2D (1D11), KIR3DL/DS1 (DX9), 2B4 (C1.7), and CD107a (H4A3) were purchased from Biolegend (CA, USA). Human LIR-1 (HP-F1) antibody was purchased from Lifespan Biosciences Inc (WA, USA). Live/Dead Aqua marker was purchased from Invitrogen (CA, USA). Flow cytometry was performed with a BD LSRII Fortessa cytometer and the data were analyzed with the FlowJo software (Treestar Inc.).

### NK Cell Transfection

A MaxCyte GT instrument (MaxCyte) was used to introduce 4 μg mRNA/1 × 10^6^ NK cells harvested 14–18 days after *ex vivo* expansion as previously reported (13). Custom-made mRNAs were obtained from TriLink Biotechnologies. Sequences that encode human CXCR4^R334X^ and CXCR4^WT^ were used (Supplementary Table 1).

### Phenotyping

*Ex vivo* expanded NK cells from six healthy donors harvested on days 14–18 were transfected with CXCR4^R334X^- or CXCR4^WT^-coding mRNA, as described above. Non-transfected NK cells from each donor were used as controls. After culturing at 37°C, 6.5% CO_2_ for 24 h, NK cells were collected and stained for 30 min at 4°C with a viability marker and cell surface markers. Cells were washed twice and fixed in 1% paraformaldehyde (MP Biomedicals) in PBS prior to acquisition on a BD LSR II Fortessa (BD Biosciences).

### Preparation of Cells for Scanning Electron Microscopy

Ten million expanded NK cells were electroporated (as described above) with either CXCR4^R334X^ mRNA or mock-transfected with vehicle alone. Non-transfected NK cells and mock-transfected (vehicle only) from the same donor were used as controls. Eight hours following transfection, 1 × 10^6^ NK cells were fixed in 4% glutaraldehyde (0.1M calcium chloride, 0.1M sodium cacodylate buffer, pH 7.2) over night at 4°C and prepared as described previously (14). Subsequently, cells were washed twice in 0.1M cacodylate and post-fixed using 1% OsO_4_ in 0.1M cacodylate buffer for 1 h. Cells were than dehydrated in an ethanol gradient series (30, 50, 70, 85, 95, and 100%). Afterwards samples were critically point dried with the Samdri-795 critical point dryer (Tousimis Research Corp, USA), mounted on aluminum stubs, and coated with 7.5 nm gold/palladium with an EMS 575-X sputter coater (Electron Microscopy Sciences, USA). Samples were imaged using the Hitachi S-3400N1 SEM at 7.5kV.

### Degranulation and Cytokine Production Assay

Expanded NK cells transfected with mRNA, as described above, were co-cultured with K562, MOLM14, SMI-LCL or MM1.S tumor cells at a ratio of 1:1 in 37°C, 5% CO_2_. After 4 h, the cells were stained with the Live/Dead viability marker, CD56 antibody, and CD107a antibody. Cells were analyzed using an LSR Fortessa II flow cytometer (BD Biosciences). In the subsequent experiments, transfected NK cells were co-cultured with K562 only for 4 h at a ratio of 1:1 in 37°C, 5% CO_2_. Brefeldin A and Monensin (BD Biosciences) and CD107a antibody were added within the first hour of co-culture. Cells were first stained extracellularly and with the Live/Dead viability marker and CD56 antibody for 30 min at 4°C before fixation and permeabilization with Cytofix/Cytoperm solution (BD biosciences). Cells were then stained for intracellular expression of TNF-α and IFN-γ in Perm/Wash buffer (BD Biosciences) for 30 min at 4°C before they were washed and analyzed using an LSR II Fortessa flow cytometer (BD Biosciences).

### *In vitro* Migration Assay

Migration assays were performed on cells 8 h after mRNA transfection using Corning Transwell® inserts. PBS containing 10% FBS and increasing concentrations of recombinant human SDF-1α (Biolegend) was added to the bottom chambers and 1 × 10^5^ NK cells suspended in the same medium but without SDF-1α were added to the top chambers. The assay plate was placed in 37°C with 6.5% CO_2_ for 2 h. Cells were collected and quantified by the CyQuant kit and assay protocol (Invitrogen). Fluorescence intensity (excitation/emission: 480 nm/520 nm) was measured on a microplate spectrometer (Infinite® 200 PRO, Tecan). Cells plated straight to the bottom chamber were used as maximum control, and the proportion of migrated cells was calculated as a percent of total cells initially added to each well. CXCR4 blockade was achieved by pretreating NK cells for 30 min with 100 μM plerixafor (Mozobil® Sanofi Oncology) in 37°C prior to the migration assay.

### Cellular Homing Assays *in vivo*

Eight to ten-week-old NOD-*scid* IL2Rgamma^null^ (NSG) mice were purchased from Jackson Laboratories. NK cells from WHIM patients or healthy donors were expanded *ex vivo* for 19 days using irradiated EBV-LCL feeders as previously described and injected intravenously (i.v.) into NSG mice at a dose of 7 × 10^6^ cells/animal. Animals received 100,000 IU of IL-2 intraperitoneally immediately following injection. Femur BM was harvested 24 h post-injection and human NK cells were identified based on CD56-positivety by flow cytometry. Ten million CXCR4^R334X^ transfected or 10 × 10^6^ non-transfected NK cells were injected i.v. into adult NSG mice. Cells were harvested from animals 24 h post-transfer from cardiac blood, liver, lungs, and the femur BM. Tissues were dissociated and analyzed by flow cytometry (Supplementary Figure 1). Human NK cells were identified based on positivity for MHC class I and human CD56.

### *In vivo* Tracking of NK Cell Using Bioluminescence Imaging

Expanded NK cells were co-transfected with either 4 μg/1 × 10^6^ cells of CXCR4^R334X^-coding mRNA and 4 μg/1 × 10^6^ cells of luciferase-coding mRNA or 4 μg/1 × 10^6^ cells of luciferase mRNA and equal volume of electroporation buffer. Eight million cells were injected i.v. into NSG mice. The biodistribution of the infused cells was tracked by bioluminescence imaging using the IVIS *In Vivo* Imaging System (PerkinElmer) 2 and 24 h post-cell infusion. Animals were injected intraperitoneally with 150 mg/kg D-luciferin Potassium salt (Gold Biotechnologies) suspended in PBS 10 min prior to imaging.

### Statistical Analysis

Data were analyzed with Prism 7.0b software (GraphPad Software Inc.), using the two-tailed paired-*T*-test, the Welch's-*T*-test, the Wilcoxon sign-rank test, or the Mann-Whitney *U*-test to report significant differences between groups. The appropriate test was chosen based on data distribution, variances, and experimental set up. ^*^P < 0.05, ^**^p < 0.01, ^***^p < 0.001, and ^****^p < 0.0001 were considered statistically significant, and ns, not significant.

## Results

### Impact of Cryopreservation, IL-2 Activation, and *ex vivo* Expansion on NK Cell Surface Expression of CXCR4 and Migration Toward the CXCR4 Ligand SDF-1α *in vitro*

**Figure 1:**
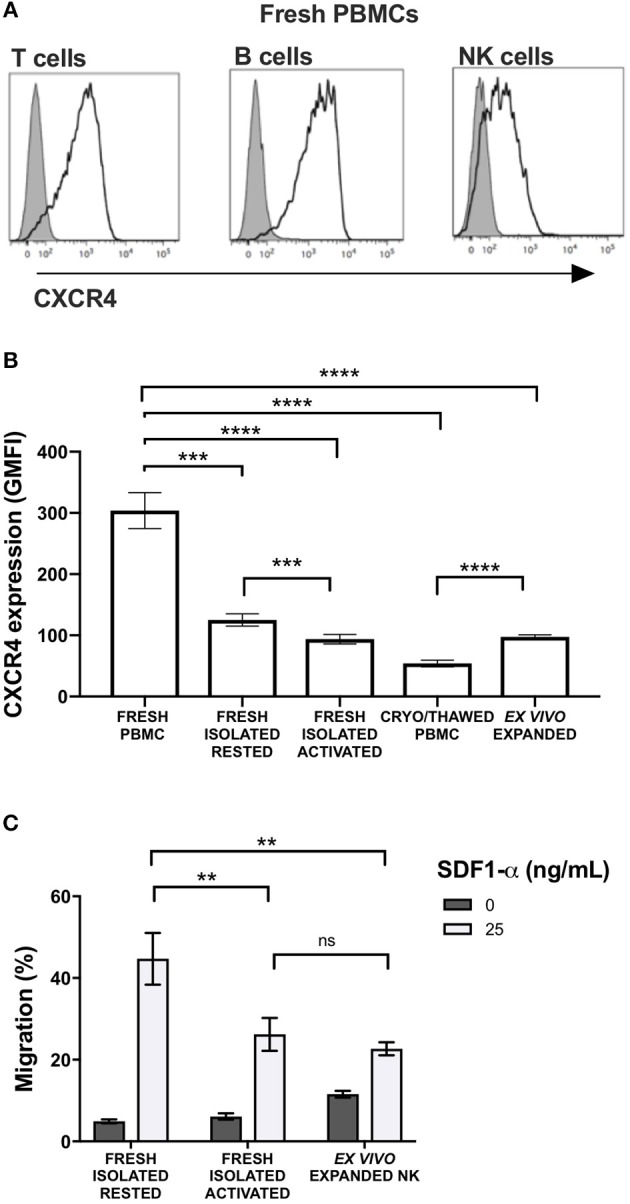
Commonly used methods to manipulate and prepare NK cells for adoptive infusion decrease surface expression of CXCR4 and reduce their ability to migrate toward SDF-1α *in vitro*. Surface expression of CXCR4 was evaluated by flow cytometry. **(A)** CXCR4 surface expression on fresh T cells, B cells and NK cells among PBMC. Representative histograms are shown (one representative donor). **(B)** CXCR4 intensity, as displayed as GMFI relative to FMO, on different NK cell preparations (*n* = 10 donors). **(C)** *In vitro* transwell migration of NK cells from populations in **(B)** toward SDF-1α (*n* = 10 donors). Statistical significance was evaluated with the paired *t*-test, two-tailed. Bar graphs present the mean and error bars report the SEM. **p < 0.01, ***p < 0.001, ****p < 0.0001.

CXCR4 was expressed on the surface of NK cells isolated from freshly collected blood samples from healthy donors, although expression levels were lower than T and B cells (Figure 1A). Given its relevance to our ongoing clinical trial evaluating the anti-tumor effects of adoptive transfer of *ex vivo* expanded NK cells, we next evaluated the impact of multiple different NK cell manipulations on CXCR4 expression including cryopreservation and thawing, IL-2 activation, and *ex vivo* expansion. Compared to NK cells within bulk PBMCs, isolated NK cells had a significant reduction in CXCR4 surface expression (*p* = 0.0002). There was an additional decrease in CXCR4 expression when isolated NK cells were rested overnight in IL-2-containing media compared to media without IL-2 (*p* = 0.003). This observation is consistent with prior reports of the effects of IL-2 on CXCR4 expression on NK, NK T, and T cells (9, 15). When freshly isolated NK cells were cryopreserved and then thawed, we observed a further substantial reduction in NK cell CXCR4 surface expression (*p* < 0.0001). *Ex vivo* expansion of the cryopreserved NK cells for 14 days using EBV-LCL feeder cells and IL-2 led to an increase of CXCR4 surface expression compared to their phenotype after thawing, however CXCR4 expression never fully recovered to the levels observed on the NK cells prior to isolation from the bulk PBMC population (Figure 1B).

Over-night IL-2 activated and *ex vivo* expanded NK cells, represents two prototypes of NK cell preparations currently being administered to humans in clinical trials (8). Compared to freshly isolated NK cells that had never been exposed to cytokines, over-night IL-2 activated and *ex vivo* expanded NK cells were less responsive to SDF-1α-induced *in vitro* migration (*p* = 0.0045 and 0.0095, respectively) (Figure 1C). As SDF-1α is known to be a critical ligand responsible for recruiting CXCR4-expressing cells into the BM (16–18), these data suggest that NK cell activation and/or *ex vivo* expansion of NK cells with IL-2 may impede the ability of these populations to home to BM compartments following adoptive transfer into patients. These data suggest reductions in NK cell CXCR4 expression that occur as a consequence of *ex vivo* manipulation approaches commonly used in most NK cell immunotherapy protocols, could have deleterious effects on this homing pathway. These observations are particularly relevant for patients who have BM-residing malignancies on clinical trials receiving adoptive NK cell infusions.

### *Ex vivo* Expanded NK Cells From Patients With WHIM Syndrome Show Enhanced *in vivo* Bone Marrow Homing Compared to NK Cells Expanded From Healthy Donors

**Figure 2:**
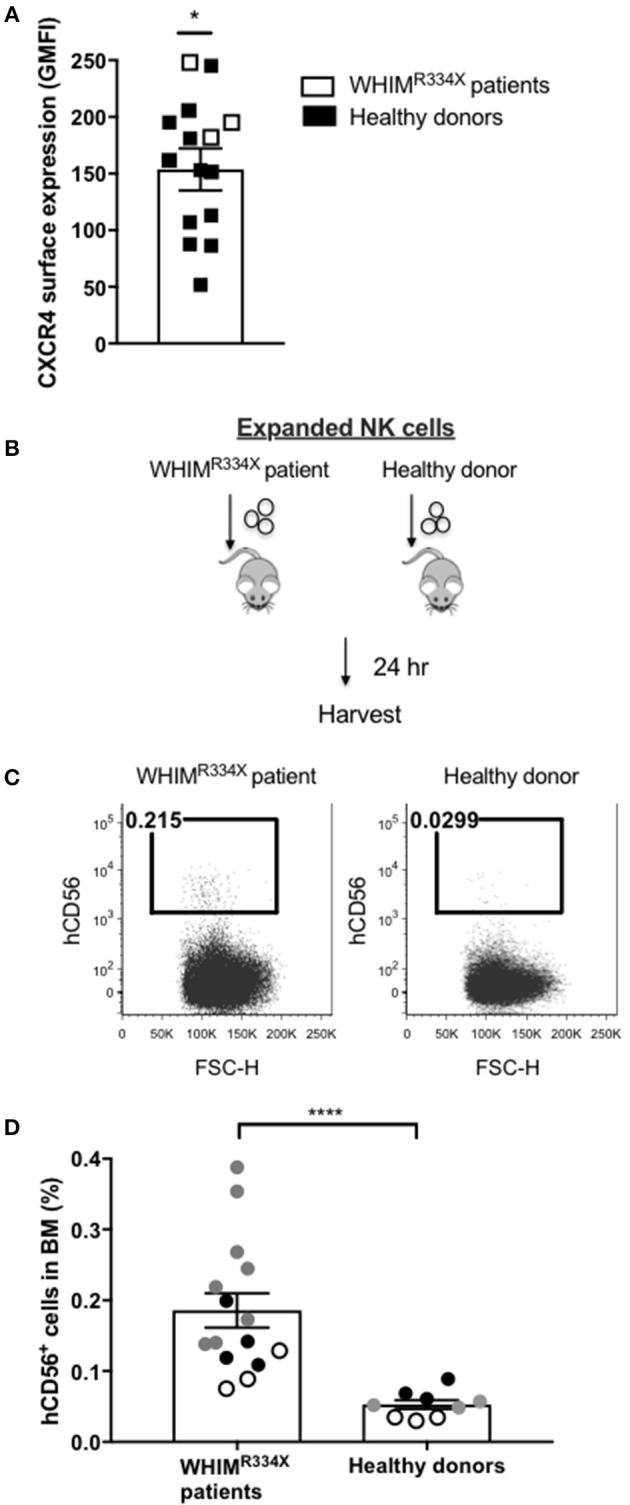
NK cells from patients with WHIM syndrome have similar intensity of CXCR4 cell surface expression but have significantly superior *in vivo* bone marrow homing compared to NK cells expanded from healthy donors. **(A)** Surface expression intensity of CXCR4 on expanded NK cells from patients with WHIM syndrome (white, *n* = 3 WHIM pts) vs. healthy donors (black, *n* = 9 donors). **(B)** Experiment schema illustrating injection of expanded human NK cells i.v. into NSG mice followed by a harvest 24 h later. **(C)** Proportion of CD56^+^ human NK cells in BM samples of mice injected with *ex vivo* expanded NK cells from healthy donors or from WHIM syndrome patients 24 h following infusion (one representative mouse from each group). **(D)** The fraction of human CD56^+^ cells recovered from the BM of mice injected with human NK cells (WHIM *n* = 15, healthy donors *n* = 9, data reported from 3 independent experiments; each experiment is represented by either white, gray or black circles). Statistical significance was evaluated by the Welch's *T*-test, two-tailed. Graphs present mean and error bars report SEM. *p < 0.05, ****p < 0.0001.

Individuals with WHIM syndrome harbor GOF mutations in the CXCR4 receptor (19), and despite having normal hematopoiesis, sequester leukocytes inside the BM niche which clinically manifests as severe leukopenia. Therefore, we hypothesized that *ex vivo* expanded NK cells expressing the GOF CXCR4 (CXCR4^R334X^) might have superior homing to the BM compared to *ex vivo* expanded NK cells expressing wild-type (WT) CXCR4. To test this hypothesis, we *ex vivo* expanded WT CXCR4-expressing NK cells from healthy donors and GOF CXCR4^R334X^-expressing NK cell from WHIM patients to evaluate the ability of the NK cells to migrate into BM compartments following adoptive transfer in immunodeficient mice. Although circulating NK cell numbers were low in the WHIM patients, we were able to successfully isolate and expanded their NK cells obtained from peripheral blood (Supplementary Table 2). The cell surface expression of CXCR4 on *ex vivo* expanded NK cells from 3 WHIM patients were higher (*p* = 0.04), although comparable with that of *ex vivo* expanded NK cells from a cohort of healthy donors (Figure 2A). Remarkably, when adoptively infused into NSG immunodeficient mice, NK cells expanded from WHIM patients had a much stronger propensity to home to BM compartments compared to NK cells expanded from healthy individuals (*p* < 0.0001) (Figures 2B–D). These data suggest strategies aimed at altering the function of the CXCR4 receptor on NK cells, rather than its overall surface density, may alone be sufficient to improve NK cell homing to the bone marrow.

### Transfection of Expanded NK Cells With CXCR4^R334X^-Coding mRNA Transiently Upregulates Cell Surface CXCR4 Leading to Improved Chemotaxis Toward SDF-1α *in vitro* Without Negatively Impacting NK Cell Viability, Phenotype, and Cytotoxic Function

We have previously shown that mRNA electroporation can be used as an approach to efficiently transfect *ex vivo* expanded NK cells using the FDA approved MaxCyte platform (11). Therefore, we explored whether mRNA electroporation of CXCR4, including the WHIM syndrome naturally occurring GOF mutated variant CXCR4^R334X^ (12), could be utilized to improve NK cell *in vitro* migration toward its ligand SDF-1α and *in vivo* homing to BM compartments in mice. We further sought to validate this strategy by testing the hypothesis that NK cells expanded *ex vivo* from WHIM patients with GOF CXCR4^R334X^ would have superior homing to the bone marrow compared to NK cells expressing wild-type CXCR4.

**Figure 3:**
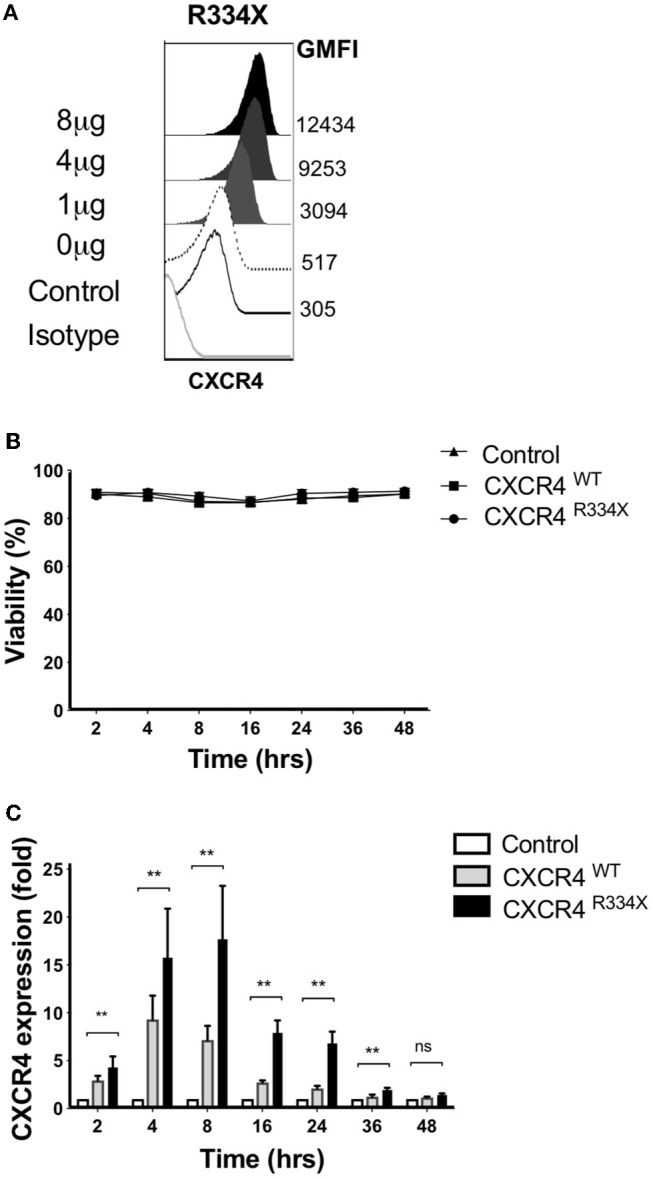
*Ex vivo* expanded NK cells transfected with CXCR4^R334X^ mRNA have significantly upregulated surface expression of the CXCR4 receptor without impacting cellular viability. **(A)** Titration of increasing amounts of electroporated CXCR4^R334X^ mRNA results in increased cell surface CXCR4 expression. **(B)** NK cell viability over time following mRNA transfection (*n* = 10 healthy donors). **(C)** CXCR4 surface expression kinetics over time reported as fold increase in CXCR4 on expanded NK cells transfected with either CXCR4^R334X^ or CXCR4^WT^ mRNA compared to their non-transfected control counterpart (*n* = 10 donors). Statistical significance was assessed by the Wilcoxon sign-rank test. Graphs present mean and error bars report SEM. **p < 0.01.

We recently reported that that mRNA electroporation using the MaxCyte GT platform can be used as an approach to efficiently transfect *ex vivo* expanded NK cells with transgenes that augment NK cell antibody-dependent cellular cytotoxicity (ADCC) and *in vitro* migration toward the CCR7 ligand CCL19 *in vitro* (11). Large scale electroporation with the MaxCyte GT instrument is clinically approved for investigational use and has been shown to be a safe and effective approach to genetically modify a variety of different cells (20–22). Therefore, we explored whether mRNA electroporation of CXCR4, including the WHIM syndrome naturally occurring GOF mutated variant CXCR4^R334X^, could be utilized to alter the expression of CXCR4 on the surface of expanded NK cells and improve their migration toward its ligand SDF-1α and *in vivo* homing to BM compartments in mice. CXCR4 mRNA electroporation efficiently augmented CXCR4 surface expression in an mRNA dose-dependent fashion (Figure 3A). Introduction of the GOF variant CXCR4^R334X^, or the wild-type protein CXCR4^WT^, did not reduce NK cell viability compared to unmodified or mock-modified cells (Figure 3B). Transfection of NK cells with either of the two mRNAs resulted in an increase of cell surface CXCR4 expression, which was transient and peaked at 4–8 h post-electroporation, then returned to baseline within 36–48 h (Figure 3C).

**Figure 4:**
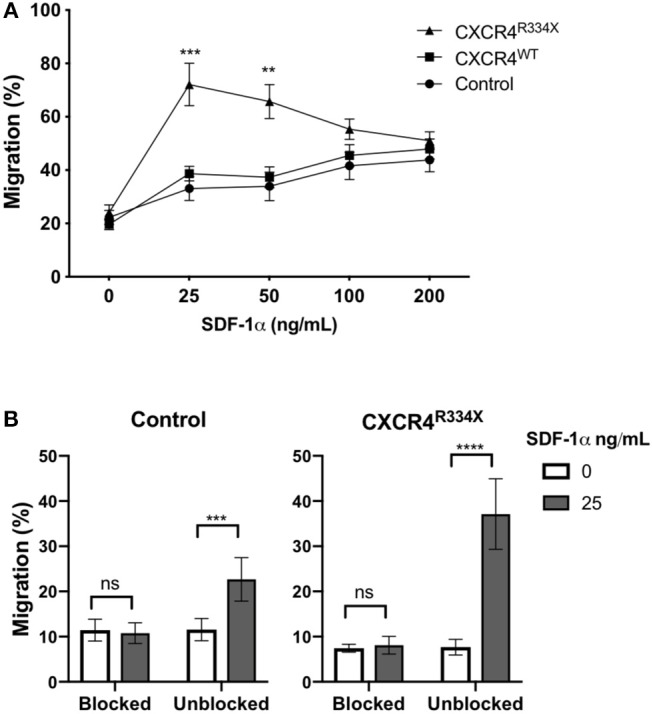
Transfection of *ex vivo* expanded NK cells with CXCR4^R334X^-coding mRNA but not with CXCR4^WT^-coding mRNA improves *in vitro* chemotaxis of cells toward SDF-1α. **(A)** *In vitro* migration of NK cells toward increasing concentration of SDF-1α (*n* = 10 donors, 4 independent experiments). **(B)** Migration toward SDF-1α when NK cells were pretreated with 100 μM of plerixafor to block the CXCR4 receptor (*n* = 9 donors, 1 experiment). Statistical significance was determined with the Student's paired *t*-test, two-tailed. Graphs present mean and error bars in panel A report SEM whereas error bars in **(B)** report SD. **p < 0.01, ***p < 0.001, ****p < 0.0001.

Expression of CXCR4^R334X^ resulted in improved *in vitro* migration toward SDF-1α (Figure 4A). Compared to both control NK cells and NK cells electroporated with CXCR4^WT^-coding mRNA, NK cells engineered to express CXCR4^R334X^ migrated more efficiently toward 25 and 50 ng/mL of SDF-1α (*p* = 0.0005 and 0.0039, respectively) (Figure 4A). A subsequent analysis revealed no differences in NK cell morphology between electroporated and non-electroporated NK cells (Supplementary Figure 2) and showed the migration potential for CXCR4^R334X^-modified NK cells, but not CXCR4^WT^-modified or unmodified cells, positively correlated with the expression intensity of cell surface CXCR4 (Supplementary Figure 3). This analysis further supports the advantage of utilizing GOF CXCR4-coding mRNA over wild-type CXCR4-coding mRNA, as the former not only results in higher cell surface expression but also appears to enhance the migration function of CXCR4 cell surface molecules to SDF-1 (Supplementary Figure 3). Importantly, we did not observe increased migration toward the non-CXCR4-binding chemokine CCL19 (Supplementary Figure 4), and pretreatment of NK cells with the specific CXCR4 agonist plerixafor abrogated the migration potential toward SDF-1α, regardless if the NK cells had undergone mRNA electroporation or not (Figure 4B). These findings confirm cellular chemotaxis assessed with this assay is dependent of the CXCR4/SDF-1α axis.

**Figure 5:**
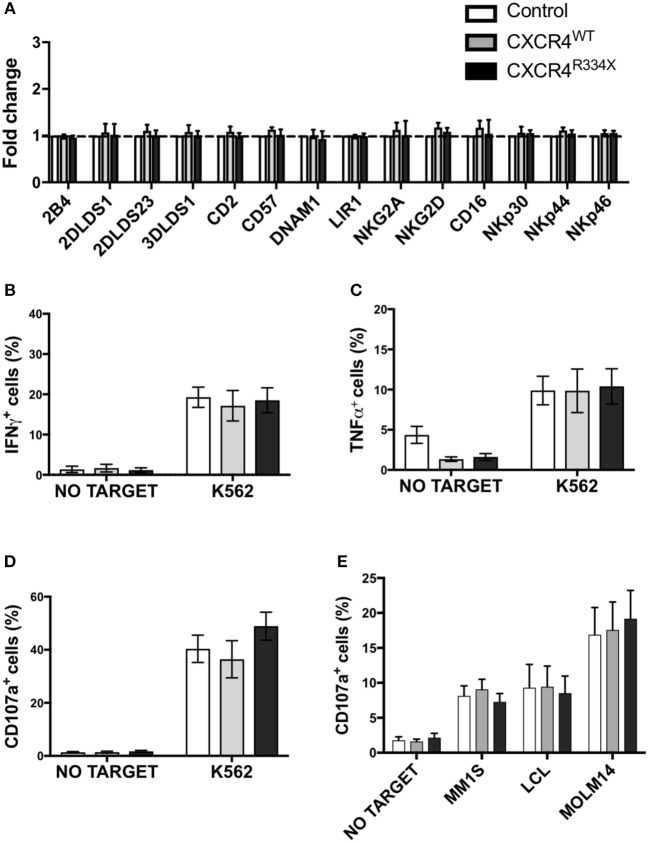
NK cells transfected with CXCR4 coding mRNAs have no alterations in NK cell inhibitory and activating receptors and have preserved anti-tumor cytotoxic function. **(A)** Inhibitory and activation NK cell receptor expression 24 h post-transfection with CXCR4-coding mRNAs compared to non-transfected control NK cells (*n* = 6 donors, 1 experiment). **(B–D)** NK cell intracellular TNF-α and IFN-γ, and surface CD107a expression following a 4-h co-culture with K562 cells (*n* = 6 donors, 1 experiment). **(E)** NK cell degranulation measured by NK cell surface upregulation of CD107a in response to co-culture with various tumor cell lines (*n* = 4 donors, 4 independent experiments). Statistical significance was determined with the Student's paired *t*-test, two-tailed. Graphs present mean and error bars report SEM.

Given the high transfection efficiency and increased *in vitro* migration of NK cells that had undergone CXCR4^R334X^ mRNA electroporation, we next conducted studies to confirm that key features of these NK cell populations, including their phenotype and cytotoxic function, were preserved. As shown in Figure 5, there was no alterations in the activating or inhibitory receptor profiles of CXCR4 mRNA transfected NK cells compared to control non-transfected NK cells (Figure 5A). Importantly, expression of other surface markers that are critical for lymphocyte homing and transmigration through endothelium such as CD62L, integrin α4 (CD49d), and CD44 were unaltered by NK cell mRNA electroporation (Supplementary Figure 5). Additionally, NK cell cytokine production capacity (IFN-γ and TNF-α) and cytotoxic function against the gold standard NK cell tumor target K562 remained unaffected by mRNA transfection (Figures 5B–D, Supplementary Figures 6, 7) (23–25). Importantly, CXCR4 mRNA electroporated NK cells had preserved high cytotoxic function against leukemia, lymphoma, and multiple myeloma cell lines as assessed by CD107a degranulation (Figure 5E). Altogether, these data demonstrate that mRNA transfection of NK cells is an effective way to transiently introduce new CXCR4 molecules on the NK cell surface, and in NK cells transfected to express CXCR4^R334X^, improve NK cell chemotaxis toward SDF-1α without negatively impacting viability, phenotype or function.

### Human NK Cells Transfected to Express CXCR4^R334X^ Have Significantly Superior *in vivo* Homing to Bone Marrow Compartments Following Adoptive Transfer Into Immunodeficient Mice Compared to Control NK Cells

**Figure 6:**
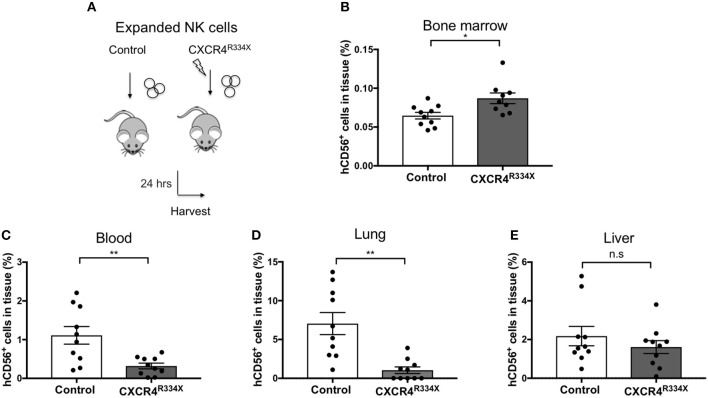
*Ex vivo* expanded NK cells transfected with CXCR4^R334X^-coding mRNA home significantly more efficiently to bone marrow compartments following adoptive infusion into NSG mice when compared to their non-transfected counterpart. **(A)** Experimental schematic illustrating the infusion of *ex vivo* expanded NK cells that have been transfected or not with CXCR4^R334X^-coding mRNA. **(B–E)** The frequency of recovered CD56^+^ human NK cells from the BM, blood, lung, and liver 24 h after infusion of the cells. Each dot represents one animal (*n* = 10 mice/group). Statistics were evaluated by the Welch's *t*-test, two-tailed. Graphs present mean and error bars report SEM. *p < 0.05, **p < 0.01.

**Figure 7:**
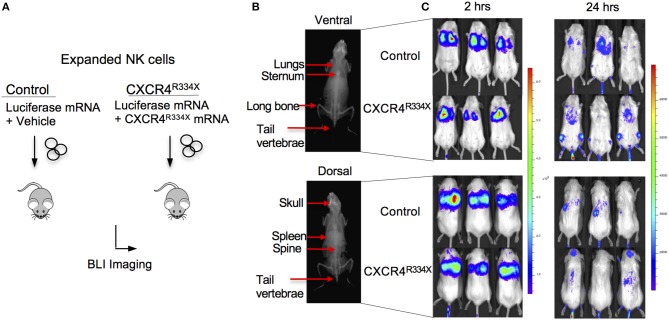
*Ex vivo* expanded NK cells transfected with CXCR4^R334X^-coding mRNA have superior trafficking to bone marrow compartments 24 h following adoptive transfer compared to their mock transfected counterpart. **(A)** Experimental schematic illustrating *in vivo* trafficking of infused NK cells transfected with mRNA coding for luciferase and CXCR4^R334X^ or luciferase alone. **(B)** X-ray image of a mouse demonstrating ventral (top) and dorsal (bottom) anatomical views of NK cell distributions observed in **(C)**. **(C)** Ventral (top) and dorsal (bottom) bioluminescence images of luciferase expressing NK cells 2 and 24 h after infusion into mice (*n* = 3 mice/group).

Because NK cells expanded from WHIM syndrome patients with CXCR4^R334X^ had superior *in vivo* homing to the BM of immunodeficient mice, and because CXCR4^R334X^ mRNA electroporated NK cells showed superior migration to SDF-1α *in vitro*, we next tested whether electroporation of CXCR4^R334X^ in expanded NK cells from healthy human donors could be used as a strategy to improve their homing to the BM in NSG mice compared to non-transfected NK cell controls. As shown in Figure 6, significantly higher proportions of CXCR4^R334X^ NK cells were recovered in the femur BM compared to controls (*p* = 0.0153). In contrast, there was a reduction in the proportion of CXCR4^R334X^ NK cells recovered in the blood and lungs compared to controls (*p* = 0.0071) (Figures 6C,D). Similar frequencies of the two infused NK cell populations were recovered from liver (Figure 6E). Altogether, these differences in biodistribution patterns are consistent with transfection of the GOF CXCR4^R334X^ receptor resulting in targeted NK cell homing to the BM. Finally, we used *in vivo* bioluminescent imaging to further confirm our findings: *ex vivo* expanded NK cells were transfected with either a combination of luciferase-coding and CXCR4^R334X^-coding mRNAs or luciferase-coding mRNA alone prior to injection into NSG mice. Bioluminescence imaging at 2 h post-infusion revealed that both cell fractions were initially trapped predominantly in the lungs, but at 24 h had assumed distinctly different anatomical localizations (Figure 7). Whereas, the mock-transfected NK cells (luciferase alone) were found in lungs, spleen, and to a low degree in the BM of the lower extremities, CXCR4^R334X^ NK cells at 24 h were found mainly in BM niches including the skull, sternum, vertebrae, and bones of the upper and lower extremities. Taken altogether, these data establish that transfection of NK cells with CXCR4^R334X^-coding mRNA enhances their ability to enter BM compartments following adoptive transfer *in vivo*.

## Discussion

The development of new methods to bolster the anti-tumor activity of adoptively infused immune cells is a pertinent topic of research within the scope of cancer immunotherapy. CAR-NK cells, checkpoint inhibition as well as bi- and tri-specific killer cell engagers are a few of the many exciting approaches that are currently being explored to improve NK cell-based immunotherapy for cancer (8). Although these and other approaches aimed at augmenting NK cell tumor killing capacity hold great promise, little research has focused on the importance, and potential for modifying NK cell homing to improve clinical outcomes. In this study, we explored mRNA transfection of NK cells to express CXCR4 (WT and CXCR4^R334X^ variants) to enhance the trafficking of *ex vivo* expanded adoptively infused NK cells into BM niches. Our data show that highly efficient transfection with the naturally occurring GOF CXCR4 variant CXCR4^R334X^ found in patients with WHIM syndrome can be achieved with electroporation using a clinically applicable platform without negatively impacting key NK cell phenotypic and functional characteristics. Establishing this method for modulating cellular homing lays the foundation for future studies exploring the therapeutic potential of adoptively infused CXCR4^R334X^-modified NK cells in patients who have BM residing malignancies.

With the specific aim of augmenting the efficacy of investigational NK cell-based cancer immunotherapies against leukemia and myeloma, we assessed the expression levels of the chemokine receptor CXCR4 on NK cells, a key receptor known to play a critical role for BM homing of multiple different cellular populations, perhaps best characterized in relation to hematopoietic stem cells (HSCs) (26–29). Further, we analyzed the impact of commonly utilized methods to isolate, process and activate NK cells on CXCR4 expression, including cryopreservation and thawing, culturing in IL-2, and *ex vivo* expansion. It is important to consider that cell handling methods and manipulation strategies used to prepare various cellular products for use in humans may have deleterious effects on the expression of cell surface chemokine receptors that play an important role in cellular homing. Nieto et al. reported ficoll treatment resulted in a decrease in expression of CCR2, CCR4, CCR5, and CXCR4 on monocytes and bulk lymphocytes (30). In this report, we show that isolation of NK cells from fresh PBMCs, as well as cryopreservation and thawing and *ex vivo* expansion leads to a significant reduction in surface expression of CXCR4. As reported previously for cytokine stimulated NK cells (9), we also observed that NK cells isolated from fresh PBMCs had a rapid reduction in cell surface CXCR4 expression following short-term IL-2 activation. As a consequence of these actions decreasing CXCR4 expression, migration capacity of NK cells toward the CXCR4 ligand SDF-1α decreased *in vitro*, which would be expected to reduce the potential of these cells to infiltrate BM compartments where malignancies such leukemia and myeloma reside. Such speculation is supported by an observation made in a recent phase I/II clinical trial of adoptive transfer of haploidentical NK cells to patients with relapsed/refractory leukemia or high-risk MDS (5). In this trial, patients treated with NK cell products that expressed high levels of CXCR4 were more likely to respond compared to those treated with NK cells expressing lower levels of this chemokine receptor. These data indirectly support the hypothesis that CXCR4 is a key receptor needed for adoptively infused NK cells to effectively target malignancies residing in the BM. Further, they establish CXCR4 as being a key candidate molecule to further study and manipulate in an effort to improve the ability of these populations to target leukemia and myeloma.

The CXCR4 receptor has long been known for its key role in chemotaxis of HSCs and other cells to the BM via signaling by its ligand SDF-1α, which is produced by BM stroma cells (31, 32). Targeted pharmacological inhibition of the CXCR4/SDF-1α interaction with plerixafor resulting in rapid mobilization of HSCs and other CXCR4-expressing cells from the BM into the circulation (33–37) and the observation that aberrant CXCR4 expression on tumor cells results in preferential BM metastasis (34, 38) highlight the singular importance that CXCR4 plays in cellular homing to the BM. The importance of this receptor in cellular trafficking to the BM was also highlighted in 2003 when GOF mutations in the CXCR4 gene were revealed as the mechanism for the sequestration of leukocytes in BM compartments of patients with WHIM syndrome (12). Since that discovery, several different families with distinct mutations in the CXCR4 gene have been described (19, 39). However, despite different mutations, WHIM syndrome patients have in common expression of CXCR4 receptors with a truncated C-terminus that impairs receptor internalization upon ligand binding and results in increased receptor signaling (39) compared to the wild type receptors (40). In this manuscript, we show for the first time that *ex vivo* expanded NK cells isolated from the circulation of patients with WHIM syndrome have stronger BM homing potential *in vivo* when infused into immunodeficient mice compared to NK cells expanded from healthy donors expressing WT CXCR4. Importantly, the observation that CXCR4 expression was not substantially more intense on NK cells expanded from WHIM patients compared to healthy controls suggest the improved BM homing capacity of these NK cells is related to augmented intracellular signaling previously shown to occur with the CXCR4^R334X^ receptor (39). The non-canonical signaling of truncated GOF CXCR4 variants is well-characterized within the context of WHIM syndrome and Waldenström's macroglobulinemia, where the hyperfunctional phenotype of truncated CXCR4 results in sequestration of cells in BM niches (12, 33, 39, 41–44).

Our study is the first to harness the potential of GOF CXCR4^R334X^ to improve the capacity of NK cells to home to BM compartments following adoptive transfer. As shown in our experiments, only the introduction of CXCR4^R334X^ mRNA lead to an improvement *in vitro* in migration to SDF-1α. Somewhat unexpectedly, we observed that electroporation of NK cells resulting in a substantial increase in surface WT CXCR4 did not augment their migration capacity to SDF-1α. These data suggest acquisition of the mutated GOF CXCR4, rather than just the level of cell surface expression is a stronger determinant for augmenting the migration capacity of CXCR4 to SDF-1α. Nevertheless, we did observe a significant correlation between CXCR4 expression intensity on CXCR4^R334X^-engineered NK cells and migration capacity, highlighting the importance of achieving nominal receptor density of this mutated receptor on genetically manipulated NK cells. Importantly, we confirmed that NK cell *in vitro* migration to SDF-1α, including the augmented migration observed to occur following introduction of CXCR4^R334X^, could be completely abrogated by blocking with CXCR4 receptor antagonist plerixafor, establishing the stand alone importance of the CXCR4/SDF-1α axis in NK cell homing and migration. Taken together, our findings suggest that the GOF properties associated with the acquisition of surface expressed CXCR4^R334X^ receptor play a key role in improving the capacity of NK cell migration toward SDF-1α. Importantly, this improvement in chemotaxis critical to BM homing following electroporation with CXCR4^R334X^ mRNA did not occur at the expense of any reduction in NK cell viability or alteration in NK cell phenotype and function.

Whereas, genetic manipulation of primary T cells has been successful for several decades, the efficacy of gene engineering NK cells has until recently been limited (10). New technologies and the optimization of well-established approaches have now allowed for the broader exploration of genetic engineering of NK cells (10). In this regard, developing approaches to direct and target NK cells to tumors and tumor-bearing tissues would be expected to complement and potentiate other strategies focused on augmenting tumor recognition and cytotoxic function.

The striking observation that expression of the CXCR4^R334X^ receptor on expanded NK cells leads to a distinct propensity toward homing to BM compartments *in vivo* following adoptive transfer into mice establishes a platform to test whether genetic manipulation to express CXCR4^R334X^ can be used as a strategy to improve NK cell killing of BM residing cancers. However, questions remain regarding optimizing the dynamics of NK cell trafficking in relation to the time dependent kinetics of surface expression of CXCR4^R334X^ receptor observed using this mRNA-based approach. Further, it is unknown whether the transient expression of CXCR4^R334X^, which lasts no longer than 48 h following mRNA electroporation, would be sufficient to mediate efficient clearance of BM-residing tumors. Nevertheless, as NK cells are naturally programmed to rapidly kill target cells without prior sensitization, the mRNA approach that we employed may provide a sufficient improvement in homing to facilitate the clearance of BM-residing tumor cells while simultaneously recruiting other critical immune cells to the tumor site (45). Depletion of receptors on the NK cell surface that compete for homing outside the BM, introduction of adhesion molecules or the use of drugs or radiation prior to cell infusion to prime BM compartments may be additional avenues explore to augment NK cell homing to BM compartments. Our data was generated using a xeno murine model to evaluate cellular homing of human cells. It is important to consider the cross reactivity of chemokine receptors with their cognate ligands may differ in humans compared to other species. Therefore, future clinical studies will need to be performed to validate our findings in humans. Finally, studies in tumor bearing animal models exploring the potential of adoptively infused NK cells genetically modified to express CXCR4^R334X^ should be conducted as they could provide insights allowing for the optimal utilization of this strategy in humans with cancer. Within this context, it will be important to exclude the possibility that the function of these genetically modified NK cells could be suppressed by mesynchymal stromal cell populations expressing high levels of SDF-1α within the BM tumor niche. Additionally, as highlighted by studies of lymphocyte trafficking into tumor infiltrated BM compartments, a potential problem with tumor-bearing animal models is the fact that immense tumor growth in the BM compartment can destroy stromal cells and suppress their ability to produce SDF-1α, dampening the potential for CXCR4-mediated migration and infiltration into the BM (46, 47). The development of models that identify the proper combination and timing of cytoreductive therapy, including chemotherapy, irradiation, and antibody therapy prior to NK cell infusion to re-establish the SDF-1α gradient could resolve or at least reduce this issue.

Collectively, data from our study suggest up-regulation of cell surface CXCR4^R334X^ on *ex vivo* expanded NK cells represents a novel approach to improve homing and target NK cell-based immunotherapies to BM where hematological malignancies reside. Importantly, our results provide a foundation for future studies aimed at bolstering the homing of NK cells to BM compartments for improved tumor eradication following adoptive NK cell infusions in patients with any BM-residing malignancies, including patients with solid tumors that reside or are metastatic to the BM. Although editing their migration potential as a stand-alone approach may improve their efficacy, combining methods that improve BM homing with strategies that augment the anti-tumor function of NK cells, may have the best chance to improve the efficacy of future adoptive NK cell immunotherapy trials in humans with cancer.

## Contribution to the Field

The processing of NK cell grafts for cancer immunotherapy often triggers reduced expression of the bone marrow homing chemokine receptor CXCR4, leading to decreased *in vitro* migration toward the chemokine SDF-1α. This perturbation may dampen the potential of adoptive NK cell therapy against bone marrow-residing malignancies such as acute myeloid leukemia and myeloma. Using an animal model, we here show that *ex vivo* expanded NK cells from WHIM patients who somatically carry the natural occurring gain-of-function variant of CXCR4, CXCR4^R334X^, have a significantly increased propensity to home into the bone marrow compared to expanded NK cells from healthy donors carrying wildtype CXCR4. Furthermore, we show that healthy donor NK cells genetically engineered using an FDA-approved transfection instrument to express CXCR4^R334X^ have significantly improved bone marrow homing potential compared to their unmodified counterparts. Our manuscript is the first proof-of-concept study to utilize gain-of-function CXCR4 to enhance bone marrow homing of primary human NK cells and will set the stage for a new line of research focusing on directing adoptively infused immune cells to the cancer. This approach is complementary to the waste majority of ongoing research focusing on improving tumor recognition and cytotoxicity of adoptively infused immune cells.

## Data Availability

This manuscript contains previously unpublished data. The name of the repository and accession number are not available.

## Ethics Statement

This study was carried out in accordance with the recommendations of the Animal Care User Committee at the NIH under protocol H-0111R1. This study was also carried out in accordance with the recommendations of Internal Review Board (IRB) at the NIH; PBMC from WHIM patient was collected at the NIH under NIH protocols NCT05-I-115 0213; NCT14-I-0185; from healthy donors under NIH protocol 99-H-0050 and under Etikprövningsnämnden (EPN) 2006/229-31/3. All subjects gave written informed consent in accordance with the Declaration of Helsinki. The protocols were approved by the IRB and EPN, respectively.

## Author Contributions

EL and MC wrote the manuscript. EL, RR, RC, and MC have designed experiments and analyzed the data. EL, FS, RR, ML, CL, and YB have performed experiments. All authors have critically reviewed the paper.

### Conflict of Interest Statement

The authors declare that the research was conducted in the absence of any commercial or financial relationships that could be construed as a potential conflict of interest.
