# Supplementary material for: Enhanced Bone Marrow Homing of Natural Killer Cells Following mRNA Transfection With Gain-of-Function Variant CXCR4R334X
Source: Front Immunol. 2019 Jun 5;10:1262. doi: 10.3389/fimmu.2019.01262 (PMC6560173; doi:10.3389/fimmu.2019.01262)
Supplement: Supplementary file 1 [file Data_Sheet_1.PDF]

Supplementary Material

1 Supplementary Figures and Tables

1.1 Supplementary Figures

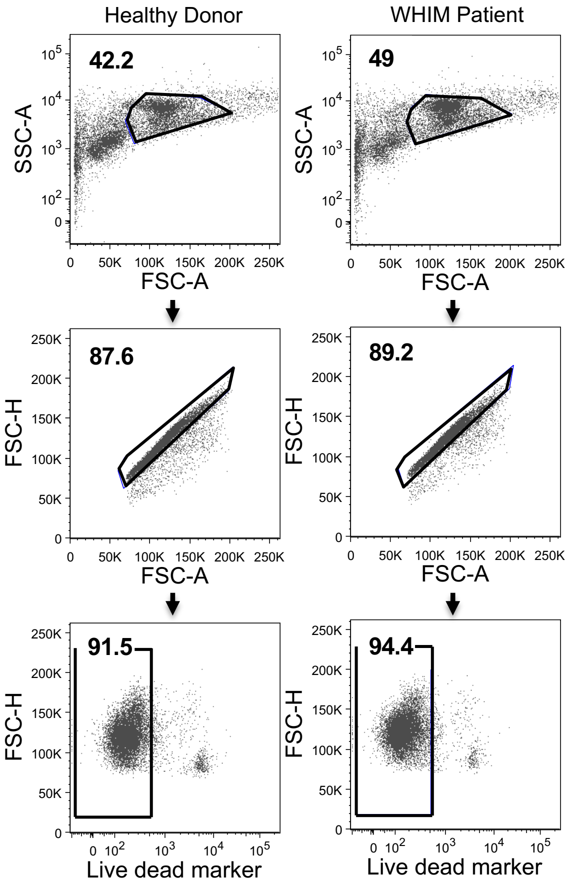

Supplementary Figure 1. NK cells *ex vivo* expanded from WHIM^{R334X} patients and healthy donors can be found in BM of mice 24 hours following infusion. Gating strategy used to evaluate BM homing of patient and healthy donor expanded NK cells 24 hours following i.v. infusion into NSG mice.

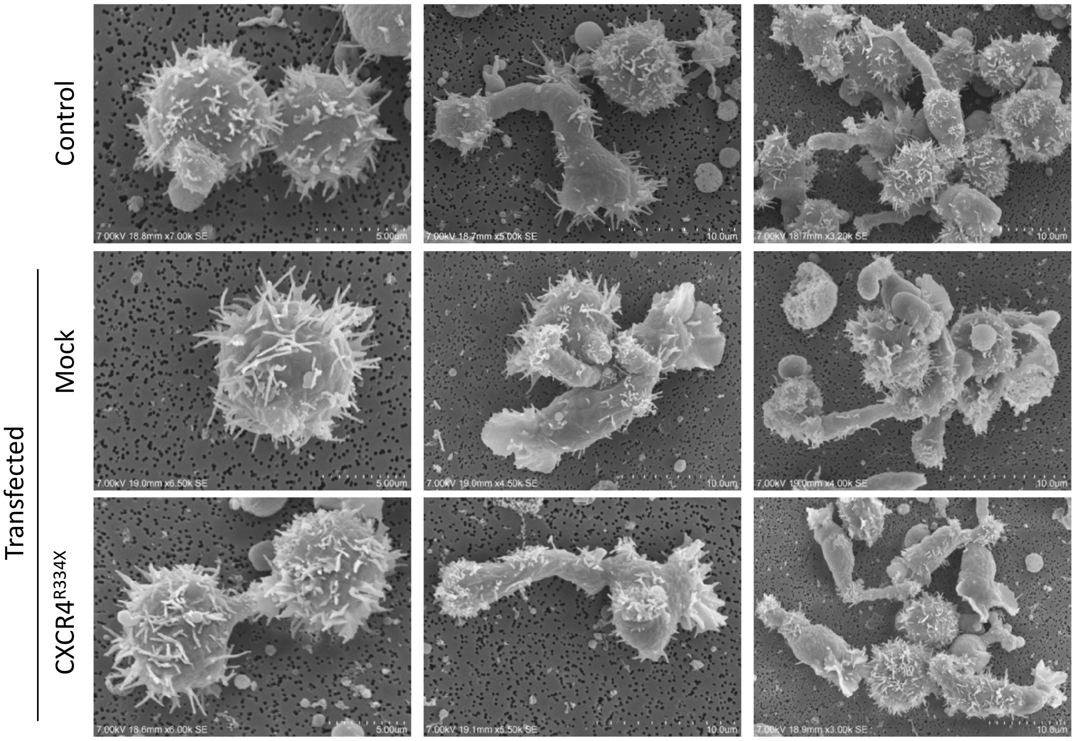

Supplementary Figure 2. **Transfection of NK cells with CXCR4^{R334X} mRNA does not change NK cell morphology.** Scanning electron microscopy images of non-transfected control NK cells and NK cells transfected with either CXCR4^{R334X} mRNA or mock-transfected with no mRNA (3 representative images per group, n=1 donor).

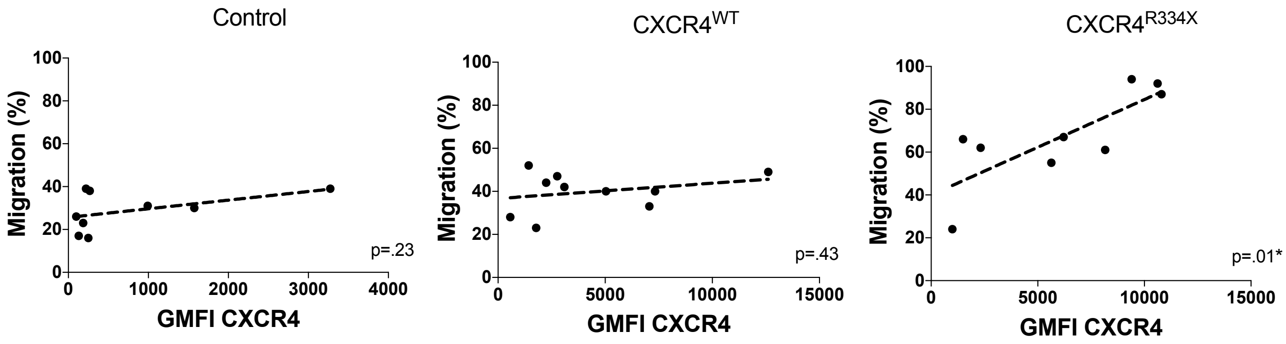

Supplementary Figure 3. Surface expression of CXCR4^{R334X} but not CXCR4^{WT} improves *in vitro* migration to SDF-1α. Correlation analysis from *in vitro* transwell migration capacity of NK cells to 25 ng/mL SDF-1α, 8 hours post transfection with either CXCR4^{WT} or CXCR4^{R334X} mRNA. Non-transfected NK cells were used as a control.

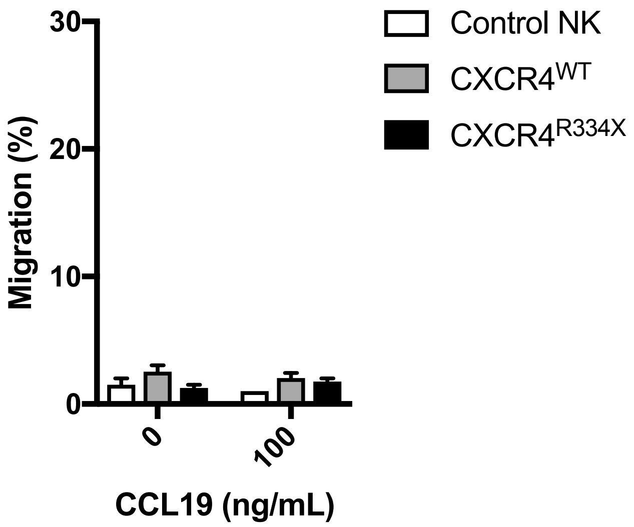

Supplementary Figure 4. CXCR4^{R334X} NK cells do not have augmented migration capacity towards the CCR7 chemoattractant CCL19. *In vitro* migration of NK cells 8 hours post transfection with either CXCR4^{WT} or CXCR4^{R334X} mRNA towards 100 ng/mL CCL19. Non-transfected NK cells were used as a control (n=4). Bar graphs present the mean and error bars report the SEM.

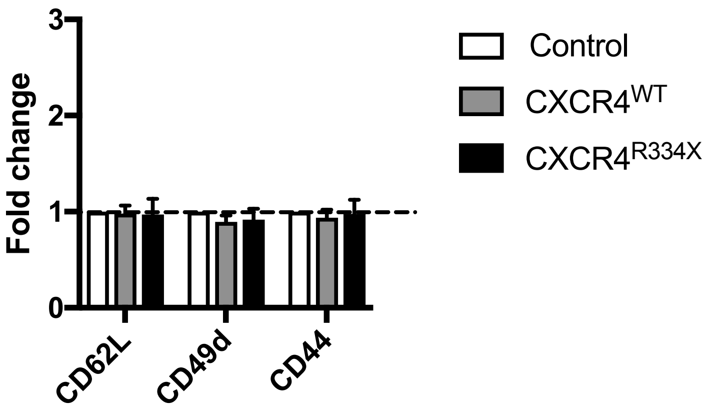

Supplementary Figure 5. NK cells transfected with CXCR4-coding mRNAs have unchanged surface expression of molecules critical for cellular adhesion and transendothelial migration. Fold change in surface expression of CD62L, CD49d, and CD44 24 hours following transfection with CXCR4-coding mRNAs compared to non-transfected control NK cells (n=6). Bar graphs present the mean and error bars report the SEM.

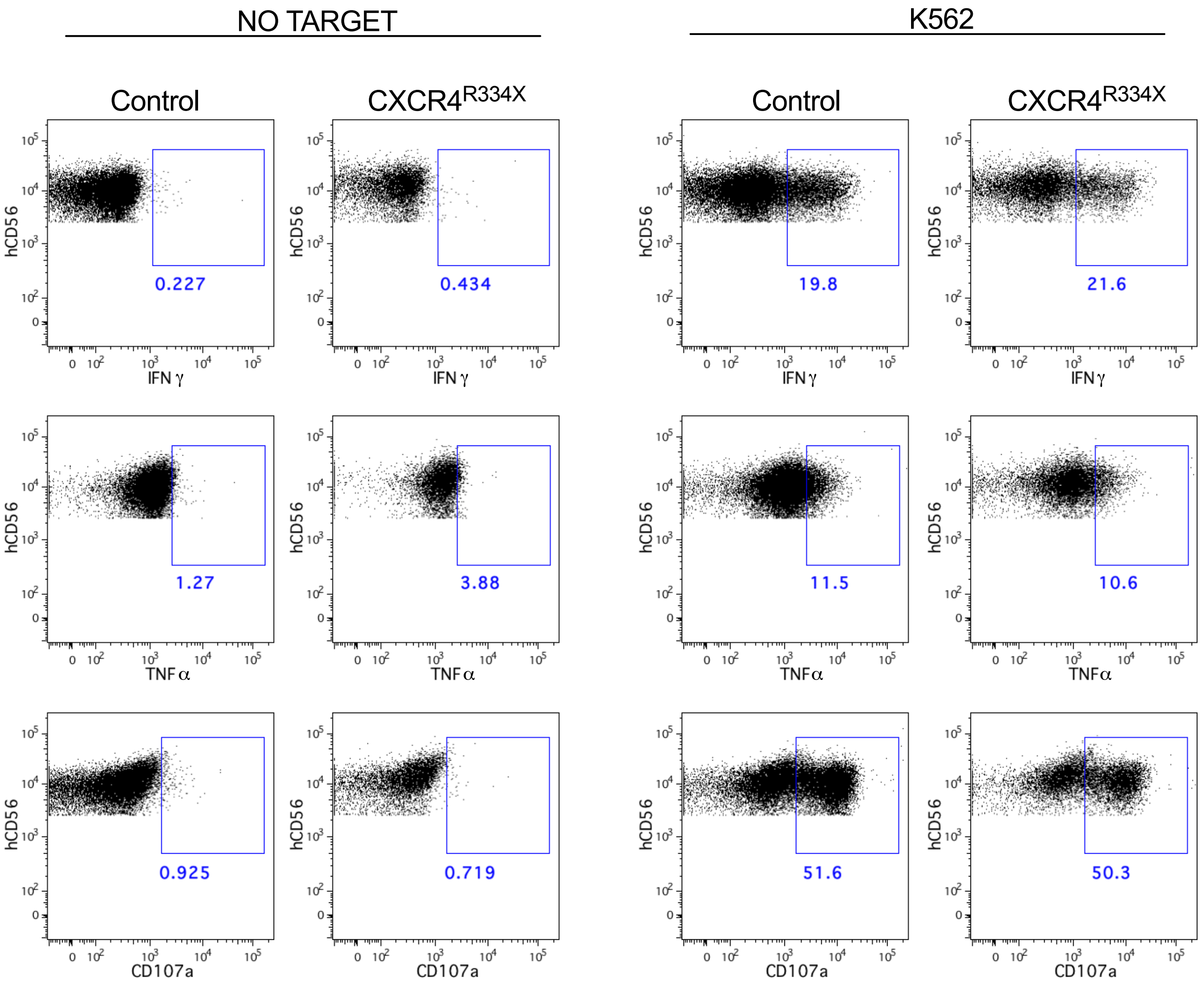

Supplementary Figure 6. Representative FACS plots for CD107a, IFN-γ and TNF-α data. Transfected and non-transfected *ex vivo* expanded NK cells were cultured alone (no target) or with K562 cells for 4 hours at a ratio of 1:1. FACS plots of cytokine production and degranulation from one representative donor are shown.

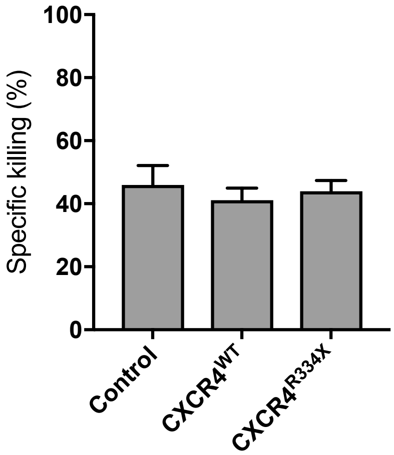

Supplementary Figure 7. Specific killing of the NK cell target K562 cells by control and mRNA transfected NK cells. Cytotoxicity of transfected *ex vivo* expanded NK cells measured by Calcien AM cell viability assay. NK cells were cultured with Calcien-labeled K562 targets for 4 hours at a ratio of 5:1 (n=3). Bar graphs present the mean and error bars report the SEM.

1.2 Supplementary Tables

Supplementary Table 1. mRNA sequences encoding the wildtype and R334X CXCR4 variants.

**CXCR4 variant**	**mRNA sequence**
Wildtype	AUGGAGGGGAUCAGUAUAUACACUUCAGAUAACUACACCGAGGAAAUGGGCUC<br>AGGGGACUAUGACUCCAUGAAGGAACCCUGUUUCCGUGAAGAAAAUGCUAAUU<br>UCAAUAAAAUCUUCCUGCCCACCAUCUACUCCAUCAUCUUCUUAACUGGCAUU<br>GUGGGCAAUGGAUUGGUCAUCCUGGUCAUGGGUUACCAGAAGAAACUGAGAAG<br>CAUGACGGACAAGUACAGGCUGCACCUGUCAGUGGCCGACCUCCUCUUUGUCA<br>UCACGCUUCCCUUCUGGGCAGUUGAUGCCGUGGCAAACUGGUACUUUGGGAAC<br>UUCCUAUGCAAGGCAGUCCAUGUCAUCUACACAGUCAACCUCUACAGCAGUGU<br>CCUCAUCCUGGCCUUCAUCAGUCUGGACCGCUACCUGGCCAUCGUCCACGCCAC<br>CAACAGUCAGAGGCCAAGGAAGCUGUUGGCUGAAAAGGUGGUCUAUGUUGGCG<br>UCUGGAUCCCUGCCCUCCUGCUGACUAUUCCCGACUUCAUCUUUGCCAACGUCA<br>GUGAGGCAGAUGACAGAUAUAUCUGUGACCGCUUCUACCCCAAUGACUUGUGG<br>GUGGUUGUGUUCCAGUUUCAGCACAUCAUGGUUGGCCUUAUCCUGCCUGGUAU<br>UGUCAUCCUGUCCUGCUAUUGCAUUAUCAUCUCCAAGCUGUCACACUCCAAGG<br>GCCACCAGAAGCGCAAGGCCCUCAAGACCACAGUCAUCCUCAUCCUGGCUUUCU<br>UCGCCUGUUGGCUGCCUUACUACAUUGGGAUCAGCAUCGACUCCUUCAUCCUC<br>CUGGAAAUCAUCAAGCAAGGGUGUGAGUUUGAGAACACUGUGCACAAGUGGAU<br>UUCCAUCACCGAGGCCCUAGCUUUCUUCCACUGUUGUCUGAACCCCAUCCUCUA<br>UGCUUUCCUUGGAGCCAAAUUUAAAACCUCUGCCCAGCACGCACUCACCUCUG<br>UGAGCAGAGGGUCCAGCCUCAAGAUCCUCUCCAAAGGAAAGCGAGGUGGACAU<br>UCAUCUGUUUCCACUGAGUCUGAGUCUUCAAGUUUUCACUCCAGCUAA
R334X	AUGGAGGGGAUCAGUAUAUACACUUCAGAUAACUACACCGAGGAAAUGGGCUC<br>AGGGGACUAUGACUCCAUGAAGGAACCCUGUUUCCGUGAAGAAAAUGCUAAUU<br>UCAAUAAAAUCUUCCUGCCCACCAUCUACUCCAUCAUCUUCUUAACUGGCAUU<br>GUGGGCAAUGGAUUGGUCAUCCUGGUCAUGGGUUACCAGAAGAAACUGAGAAG<br>CAUGACGGACAAGUACAGGCUGCACCUGUCAGUGGCCGACCUCCUCUUUGUCA<br>UCACGCUUCCCUUCUGGGCAGUUGAUGCCGUGGCAAACUGGUACUUUGGGAAC<br>UUCCUAUGCAAGGCAGUCCAUGUCAUCUACACAGUCAACCUCUACAGCAGUGU<br>CCUCAUCCUGGCCUUCAUCAGUCUGGACCGCUACCUGGCCAUCGUCCACGCCAC<br>CAACAGUCAGAGGCCAAGGAAGCUGUUGGCUGAAAAGGUGGUCUAUGUUGGCG<br>UCUGGAUCCCUGCCCUCCUGCUGACUAUUCCCGACUUCAUCUUUGCCAACGUCA<br>GUGAGGCAGAUGACAGAUAUAUCUGUGACCGCUUCUACCCCAAUGACUUGUGG<br>GUGGUUGUGUUCCAGUUUCAGCACAUCAUGGUUGGCCUUAUCCUGCCUGGUAU<br>UGUCAUCCUGUCCUGCUAUUGCAUUAUCAUCUCCAAGCUGUCACACUCCAAGG<br>GCCACCAGAAGCGCAAGGCCCUCAAGACCACAGUCAUCCUCAUCCUGGCUUUCU<br>UCGCCUGUUGGCUGCCUUACUACAUUGGGAUCAGCAUCGACUCCUUCAUCCUC<br>CUGGAAAUCAUCAAGCAAGGGUGUGAGUUUGAGAACACUGUGCACAAGUGGAU<br>UUCCAUCACCGAGGCCCUAGCUUUCUUCCACUGUUGUCUGAACCCCAUCCUCUA<br>UGCUUUCCUUGGAGCCAAAUUUAAAACCUCUGCCCAGCACGCACUCACCUCUG<br>UGAGCAGAGGGUCCAGCCUCAAGAUCCUCUCCAAAGGAAAGUAA

Supplementary Table 2. Information on the WHIM patients included in this study.

Patient	Gender	Age	Ethnicity	CXCR4 variant	$CD56^+$ cell count	Fold expansion (19 days)
1	Male	51	Caucasian	R334X	$1.08x10^5$	23
2	Female	56	Caucasian	R334X	$6.02x10^5$	66
3	Female	18	African American	R334X	$4.45x10^5$	405
